# Environmental Pollution: A Tangible Risk for NAFLD Pathogenesis

**DOI:** 10.3390/ijms141122052

**Published:** 2013-11-07

**Authors:** Mario Arciello, Manuele Gori, Roberta Maggio, Barbara Barbaro, Mirko Tarocchi, Andrea Galli, Clara Balsano

**Affiliations:** 1 Francesco Balsano Foundation, via G.B. Martini 6, Rome 00198, Italy; E-Mails: mario.arciello@fondazioneandreacesalpino.it (M.A.); manuele.gori@fondazioneandreacesalpino.it (M.G.); roberta.maggio@fondazioneandreacesalpino.it (R.M.); barbara.barbaro@fondazioneandreacesalpino.it (B.B.); 2 Department of Clinical and Molecular Sciences, Polytechnic University of Marche, Via Tronto 10, Ancona 60020, Italy; 3 Gastroenterology Unit, Department of Experimental and Clinical Biochemical Sciences, University of Florence, Viale Pieraccini 6, Florence 50139, Italy; E-Mails: mirko.tarocchi@unifi.it (M.T.); andrea.galli@unifi.it (A.G.); 4 Institute of Molecular Biology and Pathology (IBPM)-National Research Council (CNR), Piazzale Aldo Moro 7, Rome 00185, Italy

**Keywords:** environment, pollution, contaminants, particulate matter, chemicals, metals, endocrine disruptors, liver, NAFLD, nonalcoholic steatohepatitis (NASH)

## Abstract

The liver is crucial for human life, and the health of this organ often mirrors the health of the individual. The liver can be the target of several diseases, the most prevalent of which, as a consequence of development and changes in human lifestyles, is the nonalcoholic fatty liver disease (NAFLD). NAFLD is a multifactorial disease that embraces many histo-pathologic conditions and is highly linked to metabolic derangements. Technological progress and industrialization have also had the consequence of releasing pollutants in the environment, for instance pesticides or solvents, as well as by-products of discharge, such as the particulate matter. In the last decade, a growing body of evidence has emerged, shedding light on the potential impact of environmental pollutants on liver health and, in particular, on NAFLD occurrence. These contaminants have a great steatogenic potential and need to be considered as tangible NAFLD risk factors. There is an urgent need for a deeper comprehension of their molecular mechanisms of action, as well as for new lines of intervention to reduce their worldwide diffusion. This review wishes to sensitize the community to the effects of several environmental pollutants on liver health.

## 1. Introduction

Liver is a critical organ for human health; it is the center of crucial metabolic activities, and a primary line of defense against toxic compounds to which everyone is exposed daily. Liver is subjected to several types of insults and diseases, all able to alter its functions. One of the most prevalent chronic liver diseases worldwide is the nonalcoholic fatty liver disease (NAFLD), a multifactorial disease closely associated with metabolic syndrome (MeS), and considered its hepatic manifestation [1,2]. The term NAFLD encompasses a wide spectrum of liver pathologies: ranging from simple steatosis to the more aggressive form of nonalcoholic steatohepatitis (NASH), which in turn may lead to cirrhosis and, sometimes, to hepatocellular carcinoma (HCC) [3,4]. Currently, the disease progression from simple steatosis to NASH is explained by the “two-hit” theory [5]. Steatosis represents the “first hit” and makes the liver more susceptible to various “second hits”, covering a wide variety of insults and conditions such as inflammatory cytokines, oxidative stress and toxins, which cause the disease progression. Nonetheless, the underlying mechanisms of liver steatosis are still unclear [3,6]. Although metabolic derangements have been established as main risk factors for NAFLD [4], a growing body of evidence supports the idea that the exposure to some environmental factors may have a deep impact on liver diseases, including NAFLD.

Air pollution, soil and water pollutants, as well as chemicals, are acquiring increasing importance as risk factors that may contribute to the onset and progression of the disease [7–10]. Several studies in mice, human adults and children showed that air pollution can worsen the adverse effects of obesity and insulin resistance (IR), increasing oxidative stress, thus suggesting a chief role in the onset and progression of NAFLD [11–16]. However, more investigations about the effects of air pollutants on the development of NAFLD, particularly in pediatric and young individuals, are needed.

The relevance of environmental contaminants in inducing NAFLD, is underscored by the fact that in recent years the terms toxicant-associated fatty liver disease (TAFLD), and toxicant-associated steatohepatitis (TASH) have been coined to indicate the spectrum of fatty liver injury in not obese people exposed to chemicals and xenobiotics [17,18]. Furthermore, although nutritional status, co-exposures, and obesity appear to confer increased susceptibility to TAFLD/TASH [19,20], it is interesting to note that the effects of pollutants are not always linked to metabolic alterations; in fact TAFLD/TASH patients may have a low body fat mass and no IR [18].

This review article will provide a general overview of the major environmental factors and industrial chemicals, which are known to induce and/or worsen NAFLD, including novel environmental contaminants which are still under investigation.

## 2. Air Pollution: One of the Risk Factors for NAFLD

The particulate matter is often encompassed in the term “air pollution”, *i.e.*, any solid or liquid suspended in the air. The particulate is an assortment of a wide range of contaminants, it embraces smoke, vapors, smut, besides other derivatives of combustion, but it also includes sand, sea salt, spores and pollen. However, the leading role among the particulate constituents is played by the particles derived from exhausts, by-products of vaporized materials, and oxidized gases in the atmosphere such as sulfates and nitrates.

**Figure 1:**
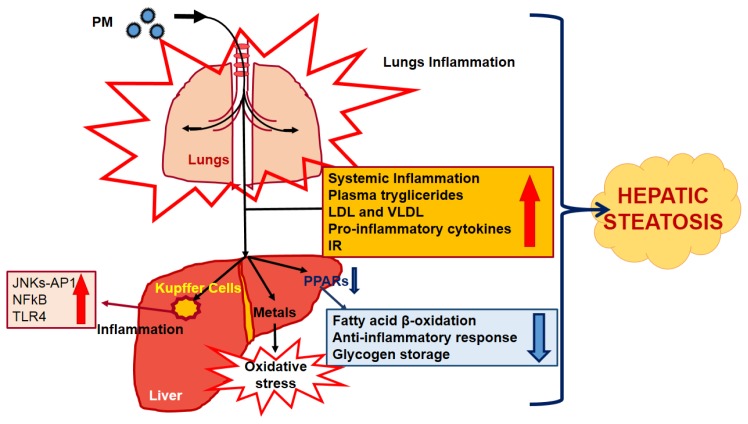
Inhaled particulate matter promotes hepatic steatosis through inflammation and several molecular and metabolic derangements. Once inhaled, PM reach the lungs alveoli where they cause inflammation. Exposure to PM is associated to systemic inflammation, rise in plasma tryglicerides, LDL and VLDL, pro-inflammatory cytokines and IR. In a secondary phase, particles arrive to the liver where they activate Kupffer cells and promote inflammation through the activation of several molecular pathways (*i.e*., JNKs-AP1, NF-κB and TLR4). Particles also affect PPARs activity, altering lipid and glucose metabolism. The actions of PM on liver could also be mediated by the metals contained within particles, whose altered content may induce oxidative stress, affecting liver health. PM: particulate matter; LDL: low-density lipoproteins; VLDL: very low-density lipoproteins; IR: insulin resistance; JNKs: c-Jun *N*-terminal kinases; AP1: activator protein 1; NF-κB: nuclear factor-κB; TLR4: Toll-like receptor 4; PPARs: peroxisome proliferator-activated receptors.

The diameter of particles, measured in micrometers (μm), is a crucial aerodynamic feature because affects their deposition and clearance by the respiratory system. In fact, particles smaller than 10 μm (*i.e*., those called PM10), are defined as inhalable. In the 1990s, it was suggested that the smallest ones—those less than 2.5 μm (PM2.5), mainly constituted by organic and elemental carbon, sulfates and nitrates [21]—are the main agents affecting human health, because they can reach the lung alveoli [22]. Based on this, scientific research has always been focused on the effects of air pollution on the respiratory system; whereas, only limited data exist about the potential role of pollution on liver. In recent years, the pathogenesis of cardiovascular diseases (CVDs) and MeS were strictly linked to the exposure to PM2.5 [14,15,23,24], indicating its deep metabolic impact. PM2.5 promotes systemic and pulmonary inflammation, prompting IR occurrence (Figure 1) [14,25,26]. It was recently demonstrated that PM2.5 exposure may synergistically act with a high-fat diet in promoting MeS [14,26], an event associated to inflammation onset that may represent a main risk factor in NAFLD progression [8]. Accordingly, mice exposed to the “real-world” PM2.5 for 10 weeks, showed inflammation, hepatic lipid accumulation, increased plasma triglycerides (TGs) and low/very low-density lipoproteins (LDL/VLDL), IR and reduced hepatic glycogen storage, with the latter representing compelling signs of an altered glucose metabolism (Figure 1) [8]. Zheng and colleagues demonstrated that a “short” exposure (3 weeks) to PM2.5 causes only a low grade lung and liver inflammation, reflecting the increase in pro-inflammatory cytokines in the plasma [8]. Nonetheless, when the exposure is prolonged to 10 weeks and PM2.5 particles reached the liver, they cause hepatic Kupffer cell activation, inducing an inflammatory response (Figure 1) [7,8,27]. This mechanism was seen to be mediated through c-Jun *N*-terminal Kinases (JNKs)-activator protein 1 (AP1), nuclear factor-κB (NF-κB) and Toll-like receptor 4 (TLR4) activation (Figure 1) [7,8]. In this pathological scenario the deregulation of peroxisome proliferator-activated receptors (PPARs) seems to play a key role [8]. In fact, PPARs are main elements in fatty acid oxidation, anti-inflammatory response and in maintaining lipid and glucose homeostasis in Kupffer cells, hepatocytes, and hepatic stellate cells (Figure 1) [28,29].

However, even if PM10 and PM2.5, known as “coarse” particles, account for the majority of the particulate matter mass, it is also true that a high percentage of particles are even smaller than 0.1 μm (PM0.1), named ultrafine particles (UFPs) [30]. The main human source of UFPs in urban settings are road vehicles, and in particular those with a diesel engine [30], involving the so-called “diesel exhaust particles” (DEP). It is known that molecules of such dimension reach the liver after inhalation [31]. It was demonstrated that in obese diabetic mice, pulmonary exposure to DEP provokes an increase in hepatic transaminases and worsens the fatty liver damage, likely through the contribution of the oxidative stress [11].

The mechanisms underlying the consequences of particulate matter (PM) inhalation are not yet known, but it is clear that the real comprehension of its effects on health can be reached only through the identification of the several constituents of these particles. However, this is a goal really difficult to reach, because PM composition can vary in different places as a consequence of the different urbanization and industrialization. Ambient PM, in fact, as mentioned above, is a mixture of a wide range of chemicals, each of which has different potential effects on health [32]. Currently, black carbon or elemental carbon (EC) is the most studied [33–36], an agent formed by the incomplete combustion of fossil fuels, biofuel, and biomass. Furthermore, it is important to underline that particulate matter also contains metals (Figure 1) [36–38]. Among them, in particular: copper (Cu), cadmium (Cd), arsenic (As) and tin (Sn) were found to have the most consistent and significant inverse associations with pulmonary functionality [39]. This correlation acquires even a greater relevance considering the fact that a prolonged exposure, as described from Zheng and colleagues [8], allows PM particles to hit the liver. This event may be crucial for NAFLD occurrence; in fact, hepatic Cu overload, as clearly demonstrated in Wilson’s disease, causes steatosis onset and progression, because this metal is able to affect the lipid metabolism and may trigger oxidative stress (Figure 1) [40]. Additionally, it is worth noting that in Wilson’s patients, PPARs seem to be important targets of Cu overload [41].

Unfortunately, besides particulate matter suspended in the air, many people worldwide, for instance chemical workers, are usually exposed to several environmental toxins that may affect the liver. These individuals may be commonly subjected to toxic inhalations; a study conducted on 25 non-obese chemical workers highly exposed to vinyl chloride (VC), revealed an 80% occurrence of TASH [20]. Despite the absence of obesity, they showed IR and a reduced adiponectin level associated to a high inflammatory state, elevated serum levels of pro-inflammatory cytokines, such as tumor necrosis factor α (TNFα), and interleukin (IL)-1β, IL-6, and IL-8. Moreover, although expressing normal serum transaminases, these subjects showed fibrosis in 55% of cases and elevated serum level of cytokeratin 18 (Ck18) [20].

Other chemicals able to affect the human metabolism are known as endocrine disrupting compounds/contaminants (EDCs). These molecules, mainly synthetic chemicals, have the ability to disrupt the endocrine system by mimicking endogenous hormones [42]. The U.S. Food and Drug Administration (FDA) created a database called endocrine disruptor knowledge base (EDKB) which is publicly available where one can find the chemical properties of a wide range of chemicals known to affect the human health [43]. Among these compounds, dioxins-polychlorinated dibenzo dioxins (PCDDs), polychlorinated dibenzo furans (PCDFs), and polychlorinated biphenyls (PCBs) need to be mentioned [10].

A recent study highlights that elevated exposure to PCBs, as well as to heavy metals, such as lead and mercury, is associated to serum alanine aminotransferase (ALT) elevation [12]. Dioxin contaminants, instead, received extensive public attention since they are known to be generated during the combustion of industrial and domestic wastes, and to escape into the environment via exhaust gases from incinerators. Matsubara *et al.* demonstrated that these molecules widely affect human health and are able to induce steatohepatitis, through the down-regulation of hepatic carboxylesterase 3 (CES3), involving the activation of inflammatory pathways, such as: transforming growth factor (TGF)-β, IL-6, signal transducer and activator of transcription 3 (STAT3) and MAD homolog 3 (SMAD) [44].

## 3. Water and Food Pollutants: The Risk to Livelihood

Being the first line of defense—and playing a pivotal role in detoxification of many drugs, hormones and environmental toxicants—the liver is the main target organ of industrial chemicals, as well as of contaminated water and food products. There are indeed several examples of drinking water and food contaminants, such as pesticides, metals (including arsenic, mercury and lead), trichloroethylene (TCE), perchloroethylene (PCE), chloroform, EDCs and many others detected in water sources and groundwater or in foodstuffs, which are known to be associated with TAFLD and TASH [10,17].

**Figure 2:**
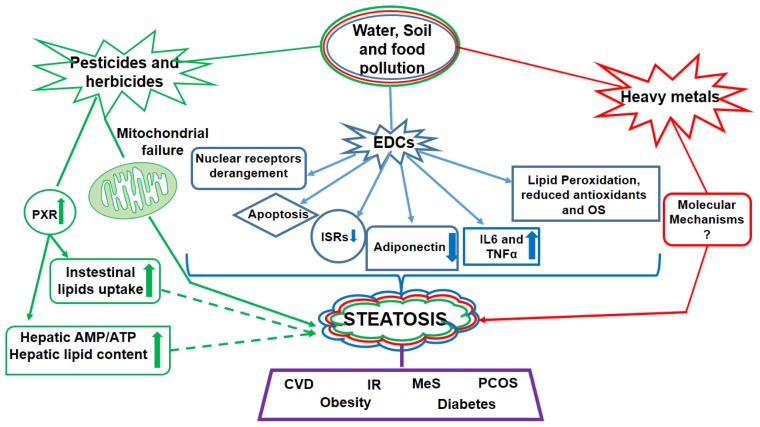
Several water, soil and food contaminants may alter metabolism through different mechanisms of action. Widely used pesticides and herbicides promote the occurrence of hepatic steatosis. To date, one potential mechanism underlying this pathological process involves the activation of the nuclear receptor PXR, which is known to promote the hepatic increase in AMP/ATP ratio and lipid content, as well as to upregulate intestinal lipids uptake (dashed lines indicate the hypothetical mechanism induced by pesticides). Pesticides and herbicides may also induce steatosis, obesity and IR by causing mitochondrial failure. Various studies reported the association of a high exposure to heavy metals (e.g., arsenic, lead, mercury and cadmium) to the incidence of NAFLD, obesity and diabetes, even though the exact mechanisms involved have not yet been elucidated. One of the most important classes of contaminants involved in a wide range of metabolic diseases is represented by the chemical compounds called EDCs. These molecules promote OS and related damage, such as lipid peroxidation, and reduce antioxidant defenses. Another consequence of the EDCs exposure is the onset of a pro-inflammatory state that modulates the expression of specific cytokines and adipokines. In fact, IL6 and TNFα were found to be higher in exposed subjects, whereas adiponectin was reduced. Strictly associated to EDCs, is the downregulation of ISRs and the occurrence of IR. At cellular and molecular levels, however, these molecules are known to induce hepatocellular apoptosis and to bind nuclear receptors (e.g., RXRs and PPARs), thus affecting their activities. This broad range of actions implies their involvement in the onset of several metabolic disorders, such as: diabetes, steatosis, obesity, MetS, PCOS and CVD. PXR: pregnane X receptor; IR: insulin resistance; ALT: alanine transaminase; EDCs: endocrine disruptor compounds; OS: oxidative stress; IL6: interleuchin 6; TNFα: tumor necrosis factor α; ISRs: insulin receptors; RXRs: retinoid X receptors; PPARs: peroxisome proliferator-activated receptors; MeS: metabolic syndrome; PCOS: polycystic ovary syndrome; CVD: cardiovascular disease.

Among pesticides and herbicides, primarily used for agricultural purposes, the triazine family induces steatosis, obesity and IR in mice, sheep and rats, by targeting mitochondrial respiration and energy production (Figure 2) [45–47]. Also other pesticides, like bendiocarb (a potent carbamate insecticide) or organophosphorus pesticides, were shown to cause steatosis in experimental animals (Figure 2) [48,49].

Considering the wide availability of these molecules, some of which are approved and recommended by the World Health Organization (WHO) for the prevention of malaria, the relevance and risk that exist for the public health become clear [50]. However, only limited data have been collected in humans and need to be increased. At molecular level, these molecules are known to bind a nuclear receptor called pregnane X receptor (PXR). This is mainly expressed in liver and intestine; it is involved in the integrity of the endocrine system and modulates the metabolism and excretion of xeno-and endobiotics [50]. In recent years, it was demonstrated that its activation might play a key role in the induction of hepatic steatosis [51,52]. Accordingly, PXR activation leads to increased hepatocellular lipid content, through increased intestinal lipid uptake and TGs synthesis, due to the up-regulation of genes involved in fatty acid uptake and mobilization [*i.e*., fatty acid translocase (CD36) and fatty acid binding protein 2 (FABP2)], and to the activation of the sterol responsive element binding protein (SREBP) [52]. The high hepatic lipid content leads to an increase of the AMP/ATP ratio [52], identified also in patients affected by hepatic dismetabolism, representing a renowned biomarker of fatty liver progression (Figure 2) [53].

Other extremely harmful environmental contaminants are heavy metals (Figure 2). Exposure to arsenic, for instance, a common contaminant of water supply, has been linked to the incidence of obesity, diabetes and NAFLD (Figure 2) in some geographical areas with significant overlap, such as in West Bengal and the USA [54–56]. However, the underlying mechanisms for this connection need further investigations. Other studies highlighted the association of lead poisoning, as well as exposure to mercury and PCBs, to the elevation of serum ALT activity in patients with suspected NAFLD, and with the occurrence of reversible micro- and macro-vesicular steatosis [12,57]. Cadmium is a common food and water contaminant [9,58]; this metal is deposited in the body, in particular in kidney and liver [9], and only a small portion is excreted per day [59]. Even if the effects of chronic cadmium exposure in liver disease are not yet known, recently, high creatinine-corrected urinary cadmium levels were positively associated with enzyme markers of hepatic necro-inflammation in NAFLD and NASH patients [9]. Interestingly, the effects of cadmium were more evident in men, who had increased risk of liver disease mortality, than in women, thus indicating even a different sensitivity to environmental pollution between the genders [9].

The main contaminants in food, water and, as we mentioned above, in the air, are the polychlorinated compounds. The most well-known for its hepatotoxic potential is probably carbon tetrachloride, also known as carbon tet, which was used in the 20th century as a dry cleaning solvent, fire extinguisher and refrigerant. This compound is able to cause a deep hepatic sufferance, so much so that it is used in animal models for the study of chronic and acute hepatic failure [60]. Nowadays, there exists a wide variety of polychlorinated compounds which are able to affect the liver. A recent analysis of the metabolic effect of PCB 153 in mice fed a high fat diet, for example, revealed that PCB 153 administration worsened metabolic changes produced by the diet, increasing steatosis and causing antioxidant depletion (Figure 2) [61]. Today, one of the main polychlorinated compounds, studied for its capacity to affect the liver, is thrichloroethylene (TCE), also known for its carcinogenic potential [62]. This compound is widely used as a solvent for metal degrease and in dry-cleaning of clothes [63], becoming over time a major environmental contaminant in soil, air, and water [64]. Furthermore, it was estimated that more than 3 million people have been directly exposed to TCE in their work [65,66]. Fatty liver associated to TCE exposure has been observed in several studies using mice [67], rats [68], and in a few cases in humans [69]. It is worth noting, that one of the most common oxidative metabolite of TCE is trichloroacetic acid (TCA), which is a ligand of PPARα, important for hepatic lipid metabolism and, if excessively activated, may participate in oxidative stress onset [18,70]. Very similar to TCE is tetrachloroethylene (PCE). In various human and animal studies PCE, a main groundwater contaminant, has been associated with liver diseases, including fatty liver [71,72]. It is indeed known that 1% to 3% of absorbed PCE metabolizes into TCA, and is sequentially eliminated in the urine [18]. Notably, in mice studies, PCE produces a similar degree of steatosis as chloroform [73]. The chloroform is a by-product of water chlorination, thus drinking water represents a source of exposure to this organic compound. Although it is known that chloroform may cause steatosis [74], the potential impact of chronic low-level environmental chloroform exposure on the fatty liver is still unknown. Thus far, the role of EDCs in the pathogenesis of NAFLD is not exhaustive, and additional epidemiological studies are needed to directly correlate these molecules to hepatic steatosis, MeS, IR and development of obesity [75–78]. However, several studies have already linked EDCs with IR and related disorders, such as polycystic ovary syndrome (PCOS) (Figure 2) [75]. The proposed pathogenetic mechanism of action of EDCs in inducing IR includes increased lipid accumulation and peroxidation, which in turn fosters oxidative stress and decreases fatty acid β-oxidation, induces insulin receptor down-regulation, increasing apoptosis and specific changes in cytokines and adipocytokines (Figure 2) [10]. Nonetheless, these human studies are only observational, and do not provide any causality, but undoubtedly justify and strongly encourage further explorations on the underlying molecular mechanisms. According to the environmental obesogen hypothesis, the inappropriate activation of nuclear receptors, such as retinoid X receptors (RXRs) and PPARγ, is known to play important roles in lipid metabolism and adipogenesis. Thus, EDC agonists, acting on some of these nuclear receptors (Figure 2), may predispose people to obesity and related metabolic disorders in conjunction with the fat-rich Western diet [79,80].

In particular, Bisphenol A (BPA), one of the most prevalent EDCs very common in food and drinks packaging, may exert a wide variety of metabolic effects. It has been shown to inhibit adiponectin production and secretion in adipocytes (Figure 2) [81], and stimulate the release of inflammatory adipokines, such as IL-6 and TNFα from human adipose tissue (Figure 2) [76]. High levels of BPA exposure, and consequent elevated urinary concentrations in adult populations, were associated with CVD, diabetes, abnormal concentrations of liver enzymes and alkaline phosphatase, as well as high oxidative stress levels, establishing a relevant contribution of BPA to IR (Figure 2) [82,83]. Moreover, elevated serum levels of BPA in women have been linked to PCOS (Figure 2) [84], suggesting an important association between PCOS and the risk of developing NAFLD, as well as with the degree of IR which rises proportionally to the development of the two pathologies [75].

Thus, all these findings and observations hint that several endocrine disruptors may take part in the development of liver steatosis, and certainly warrant further investigations.

## 4. Conclusions

Environmental pollution is a growing problem and it is reaching worrying proportions around the world. The ever-increasing energy demand linked to the urbanization and industrialization, along with economic development, led to a dramatic increase in waste discharges. In recent decades, scientists have clearly demonstrated how dangerous global environmental pollution can be for public health. It is, however, not so simple to deal with this problem because it needs to be counteracted at several levels: social, economic, and legislative. This issue may be addressed with new environmental engineering systems, as well as through an amelioration of lifestyle habits.

As already reported in the literature, pollutants may greatly affect the liver, yet scientific research on this topic is currently inadequate. For instance with regard to air pollution, even though it is comprehensible that the respiratory system is the first target to be studied, the paucity of studies regarding the liver is not justifiable as it has a critical physiological importance in body detoxification. Moreover, it has been established that the particulate matter can reach the liver under conditions of prolonged exposure, as occurs daily to all people living in large and polluted cities. Thus far, the impact of environmental contaminants on the liver has been studied, almost exclusively, only in light of their carcinogenic potential.

Environmental pollutants, such as air particulate or polychlorinated compounds, have serious and adverse effects on liver metabolism and human health; so far, such effects are seriously underestimated. However, as NAFLD is increasingly seen as a main risk factor for liver cancer development, it is desirable that the new emerging evidence about the environmental pollutant effects on liver metabolism will acquire an ever greater relevance.

This review needs to be taken into serious consideration not only by scientists, but also by public health practitioners who are often faced with cases in which liver disease is apparently of unknown origin. Unfortunately, the effects of pollutants on liver health are tangible and are not so “unknown”. As they are considered real and diffuse NAFLD etiopathogenic factors, comprehension of the molecular basis of liver damage caused by pollutants is mandatory. Thanks to the great advances recently achieved in the biotechnology field, soon it will be possible to perform detailed studies at a molecular level, involving nuclear receptors such as RXRs, PPARs or PXR that, as in “canonical” NAFLD, may play a key role in the underlying pathological mechanisms. In conclusion, the acquired knowledge can potentially have a great impact on global health management, providing new challenges for policy makers, because all of us would gain from a healthier environment.
